# Osteogenic Differentiation Capacity of Human Skeletal Muscle-Derived Progenitor Cells

**DOI:** 10.1371/journal.pone.0056641

**Published:** 2013-02-14

**Authors:** Teruyo Oishi, Akiyoshi Uezumi, Arihiko Kanaji, Naoki Yamamoto, Asami Yamaguchi, Harumoto Yamada, Kunihiro Tsuchida

**Affiliations:** 1 Department of Orthopaedic Surgery, School of Medicine, Fujita Health University, Toyoake, Aichi, Japan; 2 Division for Therapies against Intractable Disease, Institute Comprehensive Medical Science, Fujita Health University, Toyoake, Aichi, Japan; 3 Department of Orthopaedic Surgery, School of Medicine, Keio University, Tokyo, Japan; 4 Laboratory of Molecular Biology & Histochemistry, Fujita Health University Joint Research Laboratory, Toyoake, Aichi, Japan; University of Minnesota Medical School, United States of America

## Abstract

Heterotopic ossification (HO) is defined as the formation of ectopic bone in soft tissue outside the skeletal tissue. HO is thought to result from aberrant differentiation of osteogenic progenitors within skeletal muscle. However, the precise origin of HO is still unclear. Skeletal muscle contains two kinds of progenitor cells, myogenic progenitors and mesenchymal progenitors. Myogenic and mesenchymal progenitors in human skeletal muscle can be identified as CD56^+^ and PDGFRα^+^ cells, respectively. The purpose of this study was to investigate the osteogenic differentiation potential of human skeletal muscle-derived progenitors. Both CD56^+^ cells and PDGFRα^+^ cells showed comparable osteogenic differentiation potential in vitro. However, in an in vivo ectopic bone formation model, PDGFRα^+^ cells formed bone-like tissue and showed successful engraftment, while CD56^+^ cells did not form bone-like tissue and did not adapt to an osteogenic environment. Immunohistological analysis of human HO sample revealed that many PDGFRα^+^ cells were localized in proximity to ectopic bone formed in skeletal muscle. MicroRNAs (miRNAs) are known to regulate many biological processes including osteogenic differentiation. We investigated the participation of miRNAs in the osteogenic differentiation of PDGFRα^+^ cells by using microarray. We identified miRNAs that had not been known to be involved in osteogenesis but showed dramatic changes during osteogenic differentiation of PDGFRα^+^ cells. Upregulation of miR-146b-5p and -424 and downregulation of miR-7 during osteogenic differentiation of PDGFRα^+^ cells were confirmed by quantitative real-time RT-PCR. Inhibition of upregulated miRNAs, miR-146b-5p and -424, resulted in the suppression of osteocyte maturation, suggesting that these two miRNAs have the positive role in the osteogenesis of PDGFRα^+^ cells. Our results suggest that PDGFRα^+^ cells may be the major source of HO and that the newly identified miRNAs may regulate osteogenic differentiation process of PDGFRα^+^ cells.

## Introduction

Heterotopic ossification (HO) is defined as the formation of mature lamellar bone in soft tissue sites outside the skeletal periosteum. HO has been recognized to occur in many distinct contexts such as neurologic injury, trauma, and genetic abnormalities. However, the most common site is muscle and soft tissues after surgical trauma, especially total hip arthroplasty (THA) [1]. HO is diagnosed in 0.6% to 90% of patients after THA, with an average incidence of 53%. Ten per cent of patients suffer severe HO with pain in the area of the operated joint combined with a decrease in the range of motion, leading to functional impairment; [2], [3], [4], [5], [6]. Several methods for treatment of HO were reported. Nonsteroidal anti-inflammatory drugs (NSAIDs) reduce the incidence of HO when administered early (3 weeks) after spinal cord injury [7], [8], while etidronate can halt the progression of HO once the diagnosis is made if initiated fairly early (3–6 weeks) [9], [10], [11]. HO is thought to result from inappropriate differentiation of osteogenic progenitor cells that is induced by a pathological imbalance of local or systemic factors. However, the precise origin of HO has not been fully elucidated.

Skeletal muscle contains myogenic stem cells called satellite cells. Satellite cells are suggested to have the ability to differentiate into lineages other than the myogenic lineage, but a lineage-tracing study has demonstrated that they are committed to the myogenic lineage and do not spontaneously adopt nonmyogenic fates [12]. Recent studies revealed the presence of mesenchymal progenitor cells distinct from satellite cells in mouse skeletal muscle. We have identified PDGFRα^+^ mesenchymal progenitors in mouse muscle interstitium and demonstrated that these cells are responsible for fat infiltration and fibrosis of skeletal muscle [13], [14]. Interestingly, PDGFRα^+^ mesenchymal progenitors showed osteogenic differentiation in response to bone morphogenetic protein (BMP) stimulation [13], while another report showed that these cells did not differentiate into osteogenic cells when stimulated with dexamethasone, β-glycerophosphate, and ascorbic acid [15]. Wosczyna et al. recently demonstrated that Tie2^+^PDGFRα^+^Sca-1^+^ interstitial progenitors contribute to HO using a BMP2-induced in vivo bone formation model [16]. These results suggest the possibility that HO may result from PDGFRα^+^ progenitors in skeletal muscle.

MicroRNAs (miRNAs) are short, noncoding RNAs that are involved in the regulation of several biological processes including cell differentiation. It was reported that miRNAs control osteogenic differentiation. miR-138 plays a pivotal role in bone formation in vivo by negatively regulating osteogenic differentiation [17]. BMP2 treatment downregulated the expression of miR-133 and miR-135 that inhibit osteogenic differentiation by targeting Runx2 and Smad5 [18]. miR-206 inhibits osteoblast differentiation by targeting connexin 43 [19]. miR-141 and -200a are involved in pre-osteoblast differentiation in part by regulating the expression of DLX5 [20].

In this study, we isolated CD56^+^ myogenic progenitors and PDGFRα^+^ mesenchymal progenitors from human skeletal muscle and examined their osteogenic potentials both in vitro and in vivo. We also investigated miRNA expression by microarray during osteogenic differentiation of human skeletal muscle-derived progenitor cells.

## Materials and Methods

### Ethics statement

Experiments using human samples were approved by the Ethical Review Board for Clinical Studies at Fujita Health University. Muscle samples were obtained from gluteus medius muscles of patients undergoing total hip arthroplasty. Muscle samples for histological analysis were obtained from 25 patients ranging in age from 30 to 85 years with an average age of 59.5 years. Muscle samples for cell culture were obtained from 33 patients ranging in age from 35 to 82 years with an average age of 64.3 years. All patients gave written informed consent.

HO sample was obtained by thirty five-year-old man who had femoral fracture and subarachnoid hemorrhage by traffic accident and was treated by open reduction and internal fixation. X-ray analysis showed that HO was observed around the left hip joint, especially in rectus femoris muscle and vastus medialis muscle.

### Cell preparation

Muscles (typically ∼0.1–0.3 g) were placed in PBS. Nerves, blood vessels, tendons, and fat tissues were carefully removed under a dissecting microscope. Trimmed muscles were minced and digested with 0.2% type II collagenase (Worthington) for 30 min at 37°C. Digested muscles were passed through an 18-gauge needle several times and further digested for 10 min at 37°C. Muscle slurries were filtered through a 100-µm cell strainer (BD Biosciences) and then through a 40-µm cell strainer (BD Biosciences). Erythrocytes were eliminated by treating the cells with Tris-buffered 0.8% NH_4_Cl. Cells were resuspended in growth medium consisting of DMEM supplemented with 20% FBS, 1% penicillin-streptomycin, and 2.5 ng/ml basic fibroblast growth factor (bFGF) (Invitrogen) and maintained at 37°C in 5% CO_2_ and 3% O_2_.

### FACS

Cells were trypsinized and resuspended in washing buffer consisting of PBS with 2.5% FBS, and stained with PE-conjugated anti-CD56 (1∶20, Miltenyi Biotec), and biotinylated anti-PDGFRα (1∶200, R&D) for 30 min at 4°C. Cells were then stained with streptavidin-PE/Cy5 (1∶200, BD Pharmingen) for 30 min at 4°C in the dark. Stained cells were analyzed using a FACSCalibur or FACSVantage SE flow cytometer (BD Biosciences). Cell sorting was performed on a FACSVantage SE.

### Properties of human skeletal muscle-derived cells

To study the properties of human skeletal muscle-derived cells, we examined viability and population doubling time *in vitro*. To examine the viability, cells were seeded into 96-well plate (1.3×10^3^ cells/well) and incubated at 37°C in 5% CO_2_ and 21% O_2_. After 3 days, ProstoBlue reagent (Invitrogen) was added to the medium. The absorbance at 560 nm of each well was measured with the microplate reader (Thermo Fisher). To examine the population doubling time, cells were seeded into 48-well plate (3.0×10^3^ cells/well) and incubated 37°C in 5% CO_2_ and 21% O_2_. After 3 days, cells were counted, and passage into 48-well plate (3.0×10^3^ cells/well). After two more passages, the population doubling time was calculated.

### Osteogenic differentiation

Cells were seeded at a density of 4.2×10^3^ cells/cm^2^ and incubated at 37°C in 5% CO_2_ and 21% O_2_. At 50–70% confluency, osteogenesis was induced using osteogenic differentiation medium consisting of 10% FBS-αMEM supplemented with dexamethasone, ascorbate-phosphate, and β-glycerophosphate (R&D). Osteogenic differentiation medium was replaced every 3–4 days.

### Alkaline phosphatase staining

Cells were inoculated into 8-well chamber slides (3.5×10^3^ cells/well, Nalge Nunc) and cultured with osteogenic differentiation medium. At different time points, cells were washed with PBS and fixed. Alkaline phosphatase was stained using a Sigma kit #85 according to the manufacturer's protocols.

### Alizarin red S staining

For detection of calcification during osteogenic differentiation, cells were washed with PBS and fixed 100% ethanol for 8 minutes. The fixed cells were stained with 1% alizarin red S solution.

### Quantification of alkaline phosphatase activity

Cells were inoculated into 96-well plates (8.5×10^3^ cells/well) and cultured with osteogenic differentiation medium. At different time points, cells were washed with 0.9% sodium chloride and lysed in extraction solution (1% NP-40 with 0.9% sodium chloride). Alkaline phosphatase activity was assayed by a spectrophotometric method using a TRACP & ALP Assay Kit (Takara). The absorbance at 405 nm of each well was measured with the microplate reader (Thermo Fisher).

### In vivo bone formation model

To study the capacity of human skeletal muscle-derived cells to form bone, cells (1×10^6^ cells/block) were loaded onto 5 mm of PLGA-hydroxyapatite composite blocks (GC R&D Dept) and implanted subcutaneously in C.B-17/lcr-scid/scid Jcl mice (5 wks, male). As a control, the PLGA-hydroxyapatite block alone was transplanted. Implants were removed after 8 wks and fixed with 10% formaldehyde, then embedded in paraffin.

### Histochemistry and microscopy

Freshly frozen muscle tissues were sectioned (7-µm thick) using a cryostat. Fresh frozen sections were fixed with 4% PFA for 5 min. HO sample was fixed with 10% formaldehyde, then embedded in paraffin. Paraffin-embedded samples were cut into 5-µm-thick sections. For PDGFRα staining in paraffin-embedded sections, antigen was recovered using Antigen Retrival Reagent (R&D). Sections were blocked with protein-block serum-free reagent (DAKO) for 15 min, and incubated with primary antibodies at 4°C overnight, followed by secondary staining. Primary antibodies used were anti-PDGFRα (1∶200, R&D), anti-CD56 (1∶20, Miltenyi Biotec), anti-Laminin (1∶400, Sigma), and anti-lamin A/C (1∶250, Epitomics). Secondary antibodies used were Alexa Fluor 488 or 568-conjugated anti-Goat IgG (1∶1000, Invitrogen), Dylight 549-conjugated anti-mouse IgG (1∶1000, Jackson ImmunoResearch), Alexa Fluor 488 or 647-conjugated anti-Rabbit IgG (1∶1000, Invitrogen). Specimens were counterstained with DAPI (Invitrogen) and mounted with SlowFade Gold antifade reagent (Invitrogen). In some cases, specimens were subjected sequentially to Hematoxylin-Eosin (H-E) staining as previously described [13]. Stained tissues and cells were photographed with a fluorescence microscope BX51 (Olympus) equipped with a DP70 CCD camera (Olympus) or an inverted fluorescence microscope DMI4000B (Leica) equipped with a DFC350FX CCD camera (Leica). Confocal images of sections were taken using the confocal laser scanning microscope system LSM700 (Carl Zeiss).

### Microarray analysis

At different time points of osteogenic differentiation, miRNAs were purified using a miRNeasy Mini Kit (Qiagen). Microarray analysis was performed using a 3D-Gene Human miRNA Oligo chip (Toray). Extracted total RNA was labeled with Hy5 using the miRCURY LNA Array miR labeling kit (Exiqon, Vedbaek, Denmark). The hybridization was performed following the supplier's protocols (www.3d-gene.com). Hybridization signals were scanned using a 3D-Gene Scanner (Toray). The raw date of each spot was normalized by substitution with a mean intensity of the background signal determined by all blank spots' signal intensities of 95% confidence intervals. Measurements of both duplicate spots with the signal intensities greater than 2 standard deviations (SD) of the background signal intensity were considered to be valid. A relative expression level of a given miRNA was calculated by comparing the signal intensities of the averaged valid spots with their mean value throughout the microarray experiments. The microarray data were deposited in the Gene Expression Omnibus database with accession number GSE39361.

### qRT-PCR Analysis

miRNAs were extracted using miRNeasy Mini Kit (Qiagen). Reverse transcription reaction was performed using miScript Reverse Transcription Kit (Qiagen). Expression levels of miRNAs were quantified using miScript SYBR® Green PCR Kit (Qiagen). Specific primer sequences are listed below: miR-30a: UGUAAACAUCCUCGACUGGAAG, miR-146b-5p: UGAGAACUGAAUUCCAUAGGCU, miR-199b-5p: CCCAGUGUUUAGACUAUCUGUUC, miR-424: CAGCAGCAAUUCAUGUUUUGAA, miR-7: UGGAAGACUAGUGAUUUUGUUGU, miR-145*: GGAUUCCUGGAAAUACUGUUCU. U6 primer (Qiagen) was used as an internal control.

### Prediction of targets of miRNAs

Targets of miRNAs were predicted using TargetScan (http://www.targetscan.org/) and top ten targets of each miRNA were selected.

### Inhibition of miRNA during osteogenic differentiation

PDGFRα^+^ cells from three different patients were inoculated into 8-well chamber slides (3.0×10^3^ cells/well) with 10% FBS-αMEM. At 50–70% confluency, the medium was replaced to osteogenic differentiation medium without antibiotic, and the cells were transfected with 50 nM fluorescein-labeled miRCURY LNA Power Inhibitor (EXIQON) using 0.4 µl Lipofectamine (Invitrogen). For dual inhibition of miRNAs, inhibitors were used at 30 nM each. After 6 hours, the medium was changed to osteogenic differentiation medium and cells were cultured as described above. Second transfection was performed 7 days after osteogenic induction.

### Statistical analysis

Statistical significance was assessed by Student's *t* test. For comparisons of more than two groups, one-way analysis of variance (ANOVA) followed by Steel-Dwass's test was used. A probability of less than 1% or 5% (p<0.01 or p<0.05) were considered statistically significant.

## Results

### Identification of PDGFRα^+^ and CD56^+^ cells in human skeletal muscle

**Figure 1:**
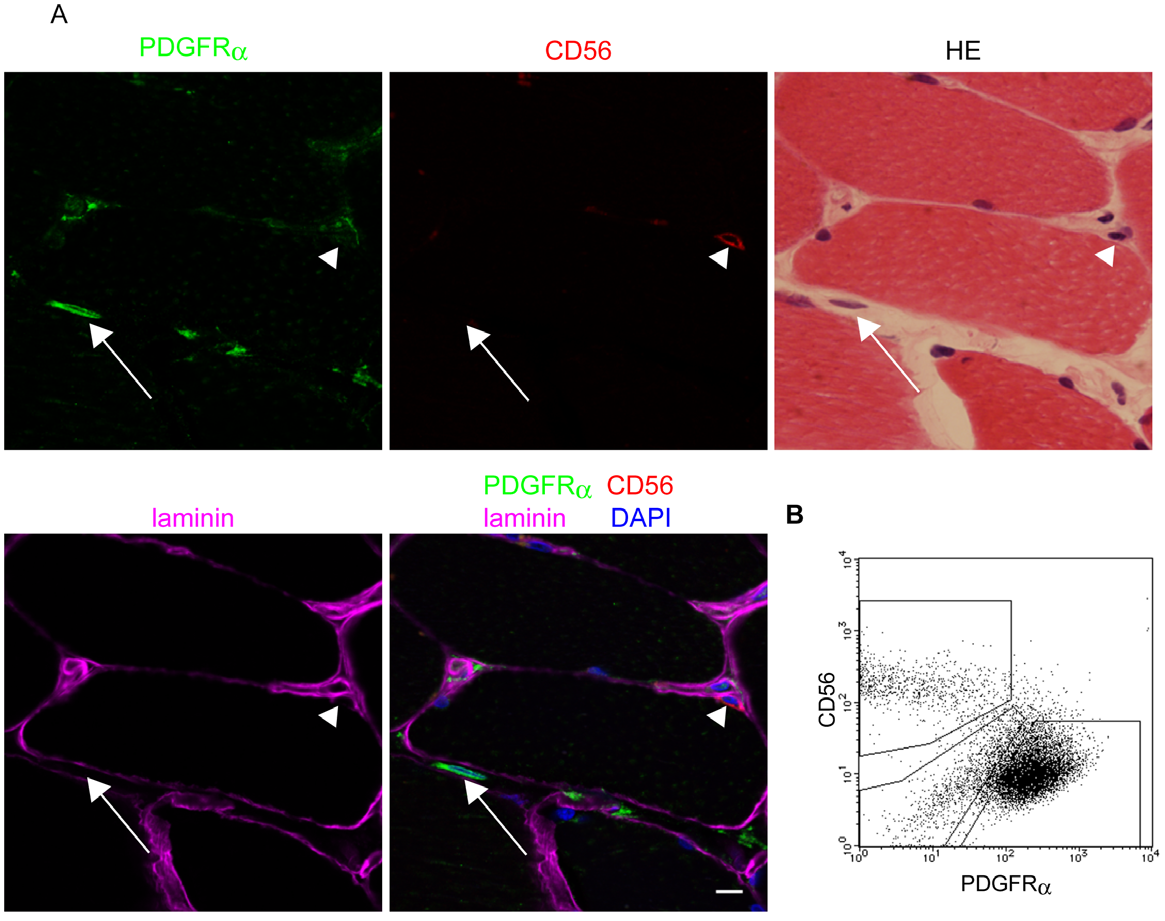
Identification of PDGFRα^+^ cells and CD56^+^ cells in human skeletal muscle. (A) Human skeletal muscle sections were stained with antibodies against PDGFRα (green), CD56 (red), and laminin (purple), and counterstained with DAPI (blue). Subsequently, specimens were subjected to H-E staining. Arrows indicate PDGFRα^+^ cells located in interstitial spaces of muscle tissue. Arrowheads indicate CD56^+^ cells residing beneath the basement membrane of myofibers. Scale bar: 10 µm. (B) Human muscle-derived cells were analyzed for the expression of PDGFRα and CD56 by FACS.

We previously identified PDGFRα^+^ cells in the interstitial spaces of mouse skeletal muscle and showed that these cells represent unique mesenchymal progenitors distinct from satellite cells [13]. Therefore, we analyzed human skeletal muscle using PDGFRα as a marker for mesenchymal progenitors and CD56 as a marker for satellite cells [21], [22], [23], [24]. PDGFRα^+^ cells were located in the interstitial spaces of muscle tissue, while CD56^+^ cells were located beneath the basement membrane of myofibers (Figure 1A). These localizations indicate that the two kinds of progenitors represent distinct populations in skeletal muscle. We next isolated these cells from human muscle. Because limited amounts of human tissue were available, we first cultured human muscle-derived cells, and then the cultured cells were subjected to fluorescence-activated cell sorting (FACS). FACS analysis also revealed that PDGFRα^+^ cells are different from CD56^+^ cells (Figure 1B). After cells were sorted, myogenic activity was detected only in CD56^+^ cells, and adipogenic activity was observed exclusively in PDGFRα^+^ cells (data not shown). These results suggest that PDGFRα^+^ cells and CD56^+^ cells are highly enriched for mesenchymal progenitors and satellite cell-derived myogenic cells, respectively. We examined basic properties of both cell populations. When cell viability was assessed, values of OD 560 nm were 0.804±0.228 for PDGFRα^+^ cells and 0.708±0.144 for CD56^+^ cells. Population doubling times were 14.00±0.52 hours for PDGFRα^+^ cells and 13.61±0.61 hours for CD56^+^ cells. There were no statistically significant differences in these properties.

### Osteogenic differentiation potential of human skeletal muscle-derived progenitors *in vitro*

**Figure 2:**
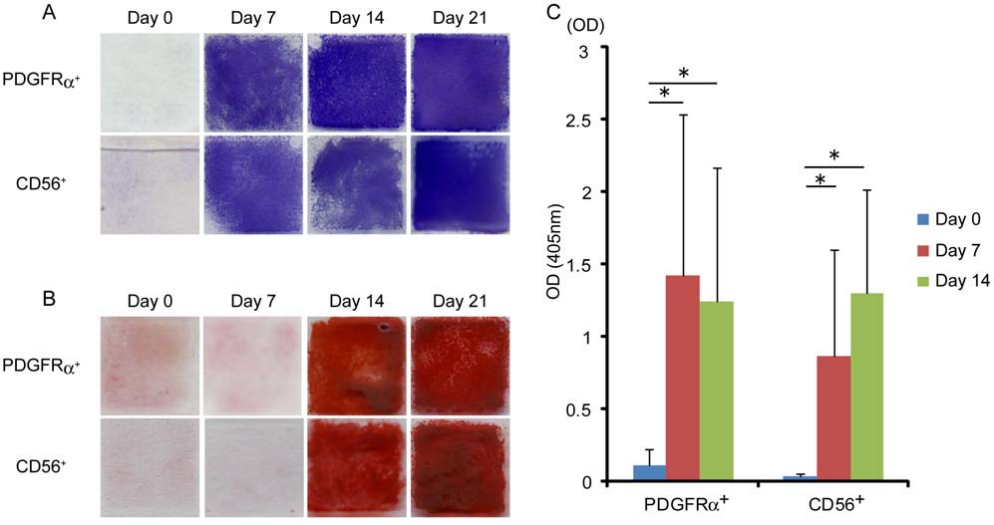
Osteogenic differentiation potential of human skeletal muscle-derived cells *in vitro*. (A) Alkaline phosphatase staining was performed at the time points indicated during osteogenic differentiation of PDGFRα^+^ cells and CD56^+^ cells. (B) Alizarin red S staining was performed at the time points indicated during osteogenic differentiation of PDGFRα^+^ cells and CD56^+^ cells. (C) Alkaline phosphatase activity of PDGFRα^+^ cells and CD56^+^ cells during osteogenic differentiation was quantified. Values are shown as means ± s.d. of ten independent preparations. *P<0.01.

Both PDGFRα^+^ cells and CD56^+^ cells were sorted and cultured under osteogenic conditions. To determine the capacity for osteogenic differentiation of human skeletal muscle-derived progenitors in vitro, we performed alkaline phosphatase (ALP) staining and alizarin red S staining. Both PDGFRα^+^ cells and CD56^+^ cells became positive for ALP at an early stage of differentiation (Figure 2A), while alizarin red S staining of both cell types did not become positive until 14 days after osteogenic induction (Figure 2B). Next, to compare the osteogenic differentiation potential of PDGFRα^+^ cells and CD56^+^ cells, we quantified ALP activity during osteogenic differentiation. After osteogenic induction, both PDGFRα^+^ cells and CD56^+^ cells showed much higher ALP activity compared with undifferentiated states. However, both cell types showed similar levels of ALP activity after differentiation, and there was no significant difference between them (Figure 2C).

### Bone-forming capacity of human skeletal muscle-derived progenitors *in vivo*

**Figure 3:**
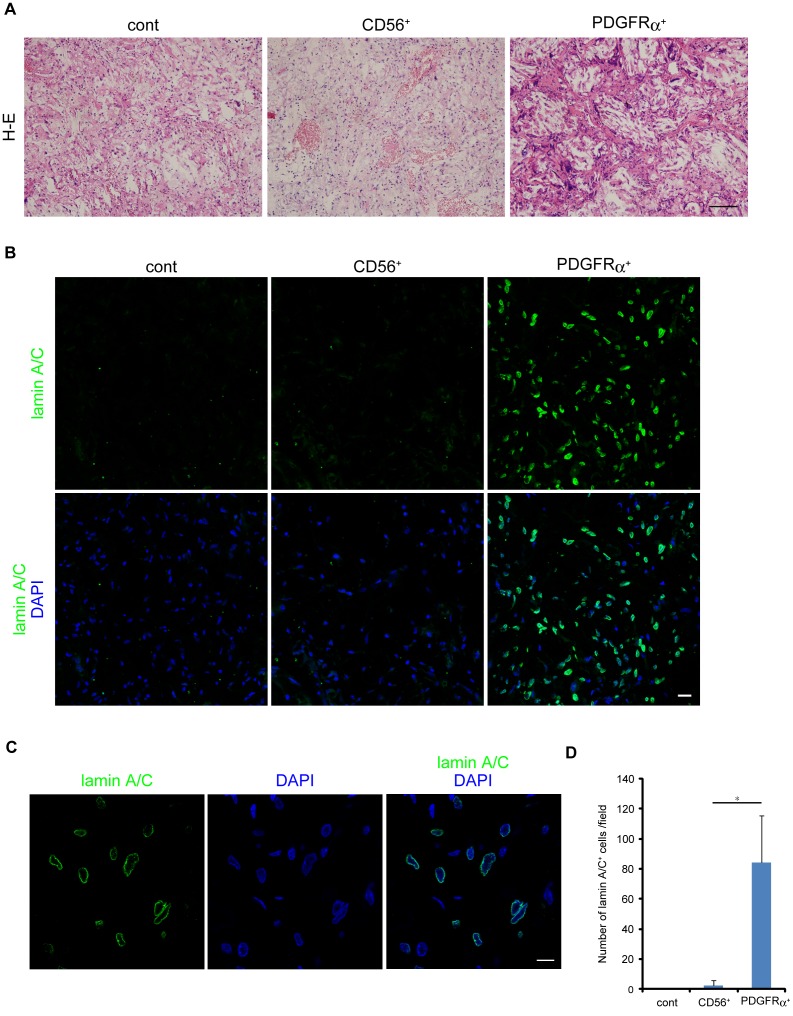
Bone-forming capacity of human skeletal muscle-derived cells *in vivo*. (A) PLGA-hydroxyapatite scaffolds containing PDGFRα^+^ cells or CD56^+^ cells were subcutaneously transplanted into immunodeficient mice. Scaffolds containing no cell were used as a control. After 8 wks, implants were removed and subjected to H-E staining. Scale bar: 50 µm. (B) Implants were stained with human lamin A/C specific antibody. Scale bar: 20 µm. (C) High magnification of PDGFRα^+^-cell transplant. Scale bar: 10 µm. (D) Human lamin-positive nuclei were counted. Number of positive cells is represented as means ± s.d., n = 15 fields from three independent implants. *P<0.01.

**Figure 4:**
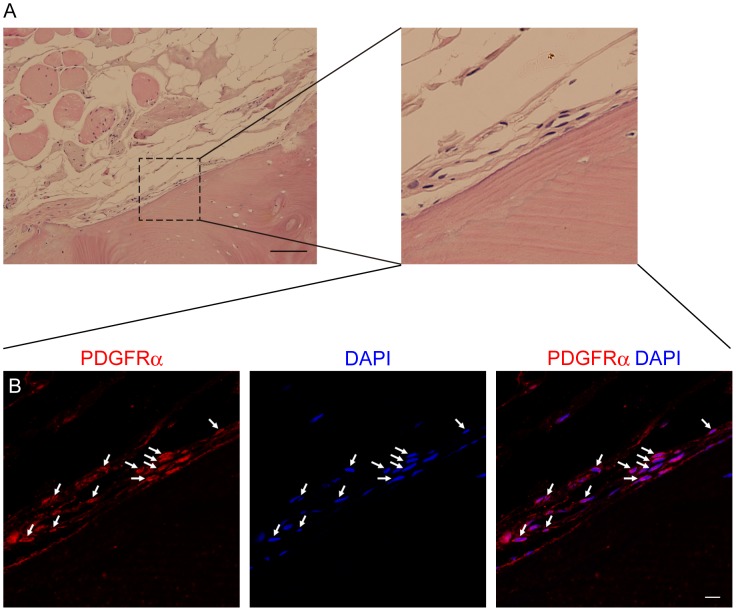
Immunohistological analysis of human HO sample. Human HO sample was subjected to immunofluorescent staining for PDGFRα and subsequently to H-E staining. (A) Image of H-E staining. Scale bar: 100 µm. Right panel shows high magnification image of square region in the left panel. (B) Image of PDGFRα staining. Arrows indicate PDGFRα^+^ cells surrounding ectopic bone tissue. Scale bar: 10 µm.

To examine the osteogenic differentiation potential of human skeletal muscle-derived progenitors in vivo, we implanted PLGA-hydroxyapatite scaffolds loaded with 1×10^6^ PDGFRα^+^ cells or CD56^+^ cells into mouse backs. This procedure is known to create an osteogenic environment in vivo (unpublished data). After eight weeks, histological analyses were performed. PDGFRα^+^ cells showed successful engraftment and had formed bone-like tissue. Structures histologically resembling medullary cavities were observed, and multinucleated giant cells were detected at the edges of cavities (Figure 3A). In contrast, CD56^+^ cells did not create such tissues (Figure 3A). To distinguish transplanted human cells from host mouse cells, we stained them with an antibody that is specific to human lamin A/C. Many human cells were detected in PDGFRα^+^ cell implants, while only a few human cells were detected in CD56^+^ cell implants (Figure 3B and D). Confocal laser microscopic analysis with high magnification revealed that nuclear envelope of human cells were clearly stained with this antibody (Figure 3C). These results suggest that PDGFRα^+^ cells possess far superior ability to adapt to an in vivo osteogenic environment than CD56^+^ cells. We also examined human skeletal muscle sample with HO in which large ectopic bone was formed within muscle tissue (Figure 4A). Intriguingly, many PDGFRα^+^ cells were found in proximity to ectopically formed bone (Figure 4A and B). In contrast, we did not observe CD56^+^ cells around ectopic bone (data not shown). Such aberrant behavior of PDGFRα^+^ cells were never observed in healthy muscle where only a few PDGFRα^+^ cells were found to be localized in the interstitial space (Figure 1A).

### Changes of miRNAs during osteogenic differentiation of human skeletal muscle-derived progenitor cells

**Figure 5:**
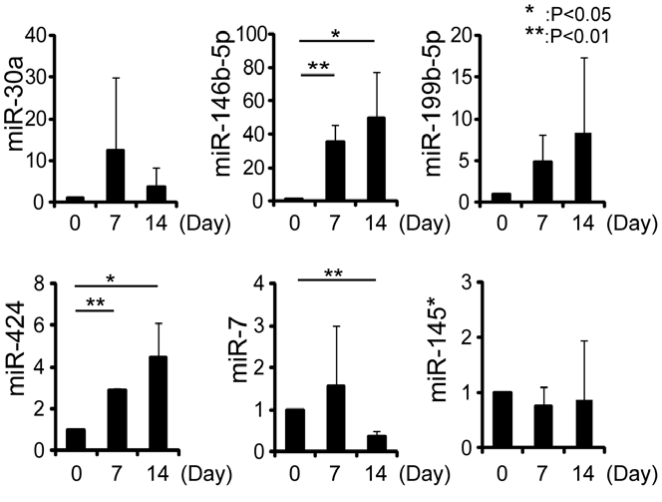
Changes in miRNA during osteogenic differentiation of PDGFRα^+^ cells. At the different time points during osteogenic differentiation of PDGFRα^+^ cells, the expressions of miRNAs indicated were quantified by qRT-PCR. Values are represented as the ratio to uninduced cells and shown as means ± s.d. of three independent preparations.

To examine the involvement of miRNAs in osteogenic differentiation of PDGFRα^+^ cells, we performed microarray analyses. We selected miRNAs that were upregulated more than twofold or downregulated to less than one half at the time points of one and two weeks after osteogenic induction. MiR-19b, -30a, -146b-5p, -199b-5p, and -424 were found to be upregulated, and miR-7, -145*, and -210 were found to be downregulated (Table 1). Interestingly, these miRNAs have not been reported in the context of osteogenesis. We confirmed the changes of these miRNAs during osteogenic differentiation of PDGFRα^+^ cells of three human different samples using quantitative RT-PCR. Upregulation of miR-146b-5p and -424 and downregulation of miR-7 were confirmed by qRT-PCR (Figure 5). We also examined the changes of these miRNAs during osteogenic differentiation of CD56^+^ cells from two different patients. miR-146b-5p and -424 tended to be upregulated but fold changes were smaller than that of PDGFRα^+^ cells, and downregulation of miR-7 were not observed (Table S1). We next predicted potential targets of these miRNAs by TargetScan. Top ten targets of three miRNAs were listed in Table 2.

**Table 1:** Changes in miRNAs during osteogenic differentiation of PDGFRα^+^ cells revealed by microarray analysis.

miRNAs	Day 7			Day 14		
	sample1	sample2	ave.	sample1	sample2	ave.
miR-19b	2.094	2.401	2.248	4.443	5.045	4.744
miR-30a	2.078	2.597	2.338	3.248	3.913	3.581
miR-146b-5p	12.255	8.533	10.394	5.692	5.161	5.427
miR-199b-5p	3.774	4.336	4.055	2.729	3.445	3.087
miR-424	2.354	2.432	2.393	3.685	3.810	3.748
miR-7	0.299	0.347	0.323	0.101	0.167	0.134
miR-210	0.161	0.204	0.183	0.083	0.102	0.093
miR-145*	0.340	0.300	0.320	0.149	0.233	0.191

Values are shown as the relative expression to uninduced cells. Day 7 and Day 14: 7 days and 14 days after osteogenic induction.

**Table 2:** Bioinformatics analysis of miRNA targets.

miRNA	Target gene	Gene name
miR-146b-5p	TRAF6	TNF receptor-associated factor 6
	IRAK1	interleukin-1 receptor-associated kinase 1
	ZBTB2	zinc finger and BTB domain containing 2
	IGSF1	immunoglobulin superfamily, member 1
	LCOR	ligand dependent nuclear receptor corepressor
	TDRKH	tudor and KH domain containing
	WWC2	WW and C2 domain containing 2
	BCORL1	BCL6 corepressor-like 1
	ZDHHC7	zinc finger, DHHC-type containing 7
	NOVA1	neuro-oncological ventral antigen 1
miR-424	FGF2	fibroblast growth factor 2 (basic)
	SLC11A2	solute carrier family 11 (proton-coupled divalent metal ion transporters), member2
	PLAG1	pleiomorphic adenoma gene 1
	ZBTB34	zinc finger and BTB domain containing 34
	MGAT4A	Mannosyl (alpha-1,3-)-glycoprotein beta-1,4-N-acetylglucosaminyltransferase, isozyme A
	PLSCR4	phospholipid scramblase 4
	ANO3	anoctamin 3
	UNC80	unc-80 homolog (C. elegans)
	TNFSF13B	tumor necrosis factor (ligand) superfamily, member 13b
	UBE2Q1	ubiquitin-conjugating enzyme E2Q family member 1
miR-7	UBXN2B	UBX domain protein 2B
	SPATA2	spermatogenesis associated 2
	C5orf22	chromosome 5 open reading frame 22
	ZNF828	zinc finger protein 828
	POLE4	polymerase (DNA-directed), epsilon 4 (p12 subunit)
	CNO	cappuccino homolog (mouse)
	RAF1	v-raf-1 murine leukemia viral oncogene homolog 1
	IDE	insulin-degrading enzyme
	EGFR	epidermal growth factor receptor
	RELA	v-rel reticuloendotheliosis oncogene homolog

### Inhibition of miR-146b-5p and -424 lead to suppression of osteogenic maturation of PDGFRα^+^ cells

**Figure 6:**
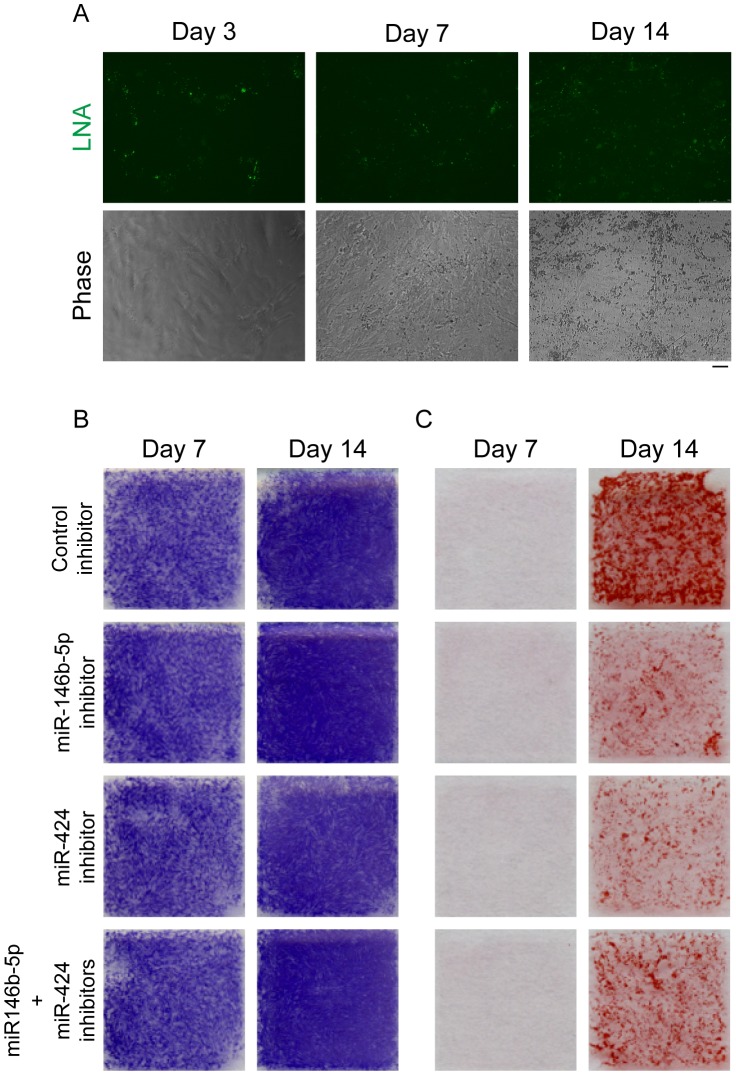
Inhibition of miRNAs during osteogenic differentiation of PDGFRα^+^ cells. PDGFRα^+^ cells were transfected with miRNA inhibitor and osteogenic differentiation was induced. PDGFRα^+^ cells transfected with control inhibitor were used as a control. Experiments were performed using cells from three independent preparations and we obtained consistent results in each experiment. Data from one representative experiment were shown. (A) Transfected fluorescein-labeled miRNA inhibitor was observed at the time points indicated. Scale bar: 50 µm. (B) Alkaline phosphatase staining was performed at the time points indicated. (C) Alizarin red S staining was performed at the time points indicated.

To clarify the role of two significantly upregulated miRNAs, miR-146b-5p and -424, in osteogenic differentiation of PDGFRα^+^ cells, we inhibited these miRNAs using LNA-based miRNA inhibitors. Transfected inhibitors were readily detectable throughout differentiation period (Figure 6A). Inhibition of these miRNAs did not affect early phase of differentiation assessed by ALP staining (Figure 6B). In contrast, alizarin red S staining revealed that inhibition of these miRNAs strongly suppressed bone matrix mineralization of PDGFRα^+^ cells (Figure 6C). Inhibitory effect of miR-146b-5p inhibitor on matrix mineralization was comparable to that of miR-424 inhibitor, and we did not see synergistic effect when both miRNAs were inhibited (Figure 6C). These results suggest that miR-146b-5p and -424 positively regulate osteogenic maturation of PDGFRα^+^ cells.

## Discussion

Although HO is thought to derive from osteogenic progenitors, these cells in human skeletal muscle have not been well characterized. Circulating bone marrow-derived osteogenic precursors are present in human peripheral blood [25], and these cells were suggested to participate in ectopic bone formation in mice and humans [26], [27], [28]. In contrast to these studies, it was also reported that there is no evidence of direct contribution of hematopoietic cells to ectopic bone formation [29], [30]. Furthermore, study of a patient with fibrodysplasia ossificans progressiva, a rare genetic disorder of heterotopic skeletogenesis, who had undergone bone marrow transplantation revealed that cells of hematopoietic origin are not sufficient to form ectopic bone [29]. Since the most common cases of HO are in muscle and soft tissues after surgery [1], it is important to understand the osteogenic properties of cells resident in skeletal muscle.

Several groups reported that presence of osteoprogenitors in human skeletal muscle. Levy et al. separated cells from human skeletal muscle into connective tissue and satellite cell fractions and found that human skeletal muscle connective tissue contained osteoprogenitor cells [31]. Mastrogiacomo et al. also isolated osteoprogenitor cells from human skeletal muscle and showed that only a few of them was positive for myogenic markers [32]. Another group isolated mesenchymal progenitor cells from traumatized human skeletal muscle [33], and suggested that these cells are the putative osteoprogenitor cells that initiate ectopic bone formation in HO [34]. In this study, we examined osteogenic properties of PDGFRα^+^ cells and CD56^+^ cells derived from human skeletal muscle. PDGFRα^+^ cells and CD56^+^ cells showed comparable differentiation potential in vitro with similar levels of ALP activity after osteogenic differentiation. However, PDGFRα^+^ cells, but not CD56^+^ cells, showed superior engraftment and bone-like tissue formation in the PLGA-hydroxyapatite scaffold-based an in vivo bone formation model. Many studies of mice showed in vitro osteogenic activity of myogenic cells. Additionally, lineage-tracing experiments have shown that Tie2-expressing cells (presumably endothelial cells) convert to the mesenchymal lineage and contribute to HO [35]. However, recent detailed analyses utilizing Tie2-Cre transgenic mice and a BMP2-induced in vivo HO model demonstrated that myogenic cells (CD31^−^CD45^−^PDGFRα^−^Sca-1^−^ cells) and endothelial cells (Tie2^+^CD31^+^ cells) do not contribute to HO, and that CD31^−^CD45^−^PDGFRα^+^Sca-1^+^ cells (named Tie2^+^PDGFRα^+^Sca-1^+^ progenitors) are the only population capable of generating ectopic bone in vivo [16]. This last study is consistent with our current study using human muscle–derived cells. Several studies have used Sca-1 as a marker to identify the nonmyogenic mesenchymal population in mouse skeletal muscle [15], [36], [37], but Sca-1 is also highly expressed in other cell types, such as endothelial cells. Furthermore, Sca-1 is not applicable to humans because there is no human homologue of Sca-1. We took advantage of the specificity of PDGFRα and identified PDGFRα^+^ mesenchymal progenitors having a superior in vivo propensity for osteogenesis in the human skeletal muscle interstitium. Taken together, PDGFRα^+^ mesenchymal progenitors in skeletal muscle can be considered to be one of the best possible candidates for the origin of HO. This notion is further supported by the fact that increased number of PDGFRα^+^ cells surround the heterotopic bone formed in skeletal muscle in human HO sample.

Interestingly, osteoprogenitor cells isolated from human skeletal muscle [32], [33] and PDGFRα^+^ mesenchymal progenitors described here possess differentiation potentials toward other mesenchymal lineages such as adipocyte and chondrocytes. Differentiation potentials toward osteogenic, adipogenic and chondrogenic lineages have been generally considered to be hallmarks of so-called mesenchymal stem cells (MSCs). Close relationship between MSCs and pericyte have been reported in several human tissues [38], [39], [40], [41]. Levy et al. also reported similarity between osteoprogenitor cells from human skeletal muscle and pericyte [31]. Crisan et al. reported more direct relationship between MSCs and pericyte. They prospectively isolated perivascular cells, principally pericytes, from multiple human tissues including skeletal muscle as CD146^+^, CD34^−^, CD45^−^, CD56^−^, ALP^+^, NG2^+^, and PDGFRβ^+^ cells [42]. Purified pericyte have shown to give rise to multipotent cells in culture that fulfill the features of MSCs. They also demonstrated that purified pericytes were readily myogenic in culture and after injection into cardiotoxin-injured or genetically dystrophic immunodeficient mouse skeletal muscle. Dellavalle et al. also reported that pericytes from human skeletal muscle possess high myogenic potential [43]. In contrast, Li et al. showed that non-myogenic cells residing in the fascia of rat or human skeletal muscle contain cells with strong chondrogenic potential [44]. Intriguingly, these chondrogenic progenitor cells lack pericyte marker CD146. Similarly, PDGFRα^+^ mesenchymal progenitors described here have little myogenic potential and are distinct from pericytes. Lecourt et al. also reported that only CD56^+^ fraction from human skeletal muscle has myogenic potential and only CD56^−^ fraction show adipogenesis [45]. Further studies are required to completely understand the relationship between MSCs and pericyte, and their myogenic potential.

We identified three miRNAs that show dramatic changes during osteogenic differentiation of PDGFRα^+^ cells. Although these miRNAs have not been reported in the context of osteogenesis, we showed that matrix mineralization was significantly suppressed by the inhibition of two upregulated miRNAs, miR-146b-5p and -424. Since miR-146b-5p and -424 have distinct sequence in their seed region, they may target different set of mRNAs. However single inhibition of each miRNA and dual inhibition of two miRNAs resulted in comparable suppression of mineralization (Figure 6C). These results suggest that miR-146b-5p and -424 stimulate osteogenic maturation of PDGFRα^+^ cells by inhibiting different targets lying within the same signaling pathway.

TargetScan predicts TNF receptor-associated factor 6 (TRAF6) and interleukin-1 receptor-associated kinase 1 (IRAK1) as the targets of miR-146b-5p (Table 2). TRAF6 and IRAK1 are key adapter molecules in the NF-κB signaling pathway. NF-κB activation requires the phosphorylation and degradation of inhibitory κB (IκB) proteins, which is induced by IκB kinase α (IKKα) and IKKβ. TRAF6 and IRAK1 mediate IKK activation, thereby lead to NF-κB activation [46]. TRAF6 and IRAK1 have been demonstrated to be the direct targets of miR-146b-5p and therefore miR-146b-5p inhibit NF-κB signaling by targeted repression of TRAF6 and IRAK1 [47], [48], [49]. Interestingly, TargetScan also predicts IRAK2 and IKKβ as the targets of miR-424, although they are not included in top ten targets shown in Table 2. Human IRAK2 has no splice variants, while mouse has four IRAK2 isoforms generated by alternative splicing at the 5′-end of the gene and two of which are inhibitory [50]. Thus IRAK2 in human and mouse may function differently. Importantly, recent study showed that IRAK2 is required for NF-κB activation in human cells [51]. Although NF-κB pathway has been well studied in osteoclasts, recent studies also revealed its importance in osteoblasts as a negative regulator of bone formation [52], [53]. Taken together, miR-146b-5p and -424 may inhibit NF-κB signaling by targeting different signal mediators such as TRAF6, IRAK1, IRAK2 or IKKβ, and exert a stimulatory effect on osteogenesis of PDGFRα^+^ cells.

In conclusion, we isolated human skeletal muscle-derived progenitor cells and characterized their osteogenic properties. Our results suggest that PDGFRα^+^ mesenchymal progenitors residing human skeletal muscle are the major origin of HO. In addition, the miRNAs analysis presented here may provide useful information to understand the mechanism whereby miRNAs regulate osteogenic differentiation of PDGFRα^+^ mesenchymal progenitors.

## Supporting Information

Table S1 (DOC)
