# Supplementary material for: Osteogenic Differentiation Capacity of Human Skeletal Muscle-Derived Progenitor Cells
Source: PLoS One. 2013 Feb 14;8(2):e56641. doi: 10.1371/journal.pone.0056641 (PMC3572948; doi:10.1371/journal.pone.0056641)
Supplement: Table S1 — (DOC) [file pone.0056641.s001.doc]

Table S1. Changes in miRNAs during osteogenic differentiation of CD56^+^ cells examined by qRT-PCR

| miRNAs | Day 7 | | | Day 14 | | |
| --- | --- | --- | --- | --- | --- | --- |
|  | sample 1 | sample 2 | ave. | sample 1 | sample 2 | ave. |
| miR-30a | 3.340 | 1.158 | 2.249 | 0.490 | 1.209 | 1.699 |
| miR-146b-5p | 4.408 | 1.045 | 2.727 | 4.378 | 2.042 | 3.210 |
| miR-199b-5p | 1.240 | 2.615 | 3.855 | 2.651 | 6.119 | 4.385 |
| miR-424 | 4.868 | 1.643 | 3.256 | 2.479 | 1.910 | 2.195 |
| miR-7 | 1.209 | 0.574 | 0.892 | 1.640 | 0.905 | 1.273 |
| miR-145* | 4.970 | 1.819 | 3.395 | 1.892 | 2.688 | 2.290 |

Values are shown as the relative expression to uninduced cells. Day 7 and Day 14: 7 days and 14 days after osteogenic induction.
